# A Microfluidic Chip for Liquid Metal Droplet Generation and Sorting

**DOI:** 10.3390/mi8020039

**Published:** 2017-01-27

**Authors:** Lu Tian, Meng Gao, Lin Gui

**Affiliations:** Key Laboratory of Cryogenics, Technical Institute of Physics and Chemistry, Chinese Academy of Sciences, 29 Zhongguancun East Road, Haidian District, Beijing 100190, China; lutian@mail.ipc.ac.cn (L.T.); mgao@mail.ipc.ac.cn (M.G.)

**Keywords:** microfluidics, liquid metal, droplets, dielectrophoresis

## Abstract

A liquid metal based microfluidic system was proposed and demonstrated for the generation and sorting of liquid metal droplets. This micro system utilized silicon oil as the continuous phase and Ga_66_In_20.5_Sn_13.5_ (66.0 wt % Ga, 20.5 wt % In, 13.5 wt % Sn, melting point: 10.6 °C) as the dispersed phase to generate liquid metal droplets on a three-channel F-junction generator. The F-junction is an updated design similar to the classical T-junction, which has a special branch channel added to a T-junction for the supplement of 30 wt % aqueous NaOH solution. To perform active sorting of liquid metal droplets by dielectrophoresis (DEP), the micro system utilized liquid-metal-filled microchannels as noncontact electrodes to induce electrical fields through the droplet channel. The electrode channels were symmetrically located on both sides of the droplet channel in the same horizontal level. According to the results, the micro system can generate uniformly spherical liquid metal droplets, and control the flow direction of the liquid metal droplets. To better understand the control mechanism, a numerical simulation of the electrical field was performed in detail in this work.

## 1. Introduction

Droplet-based microfluidics, which is also called “digital microfluidics”, has recently become a hot spot in a lot of chemical [1] and biological [2,3,4] sciences. This system has many unique and fundamental features, such as the ability to handle small volumes of fluid, throughput repeatability, low unit cost and so on. These features allow a lot of research processes to be performed in a cheaper, faster and more efficient way [5].

As a special fluid, room temperature liquid metal (typically gallium-based alloys) has also attracted much attention in microfluidics as a unique material in flexible electronics [6] and micro devices [7]. EGaInZn, a less volatile material, is safe to the human body and biocompatible for even implantable medical uses. Its other features, such as large surface tension, flowability, low heat capacity, high thermal and electrical conductivity [8], also make it a very unique material for many special applications [9,10,11]. Combining advantages of both droplet and liquid metal, liquid-metal-droplet-based microsystems have a lot of potential uses in heat transfer and drug delivery applications.

Normally on-chip microfluidic droplets are generated by utilizing two immiscible fluids as two phases: continuous phase and dispersed phase. Typically, these two phases intersect at one point. Then, the dispersed phase is sheared off by the continuous phase at a T-junction or flow-focusing microchannel [12,13]. However, the traditional droplet generation methods cannot be directly used for gallium-based alloys droplet generation, because the gallium-based alloys have very large surface tension and become sticky when oxidized easily. Koo et al. [14] manipulated liquid metal droplets utilizing water, mineral oil or hydrocal 2400 naphthenic base oil as the carrier liquid by tunable radio frequency. They observed that a large number of EGaIn oxide layers remained on the microchannel walls. Dickey et al. [15] found that the outer oxide layer strongly protected the liquid metal from shape changing. Some research groups demonstrated that EGaInSn liquid metal, enfolded by this oxide layer, could even keep an irregular shape [16,17]. Hutter et al. [18] used polyethylene glycol (PEG) and N_2_ treated silicon oil to reduce dissolved O_2_ to prevent gallium oxidation. However, this method is very complicated and may block microchannel. Li et al. [19] demonstrated a system to reduce oxidized Galinstan. They placed two HCl-filled microchannels on both sides of the Galinstan microchannel. The HCl molecules penetrated through the polydimethylsiloxane (PDMS) wall by diffusion and reduced the surface oxidation of Galinstan inside. However, this method could only reduce the oxidation and the effect was limited because of the slow diffusion process. Hence, it is necessary to develop a simpler, cheaper and more efficient method for liquid metal microdroplets generation without oxidation.

The manipulation of droplets is another problem in the droplet-based microfluidics. Usually, it can be divided into two categories: passive methods and active methods. The passive method often utilizes the characteristics of droplets themselves and channel geometry to manipulate droplets. Tan et al. [20] designed a useful and efficient microfilter to sort droplets by sizes. However, the sorting criteria of the passive method cannot be changed once set; the passive methods always lack flexibility. The active manipulation methods have more advantages in complicated applications, because their control strategy can be changed or planned as desired at any time. The active manipulation of droplets has been widely studied by many groups in recent years [21,22,23]. Kumar et al. [24] used the optoelectric technique to manipulate water droplets. This method can hardly be performed on liquid metal droplets, because the light cannot pass through liquid metal. Dielectrophoretic control is another important active droplet manipulation method, especially for neutral droplets. Due to its short response time, good compatibility with microfluidic systems, the dieletrophoresis (DEP) effect has been used to manipulate particles, cells and viruses [25,26] in micro/nano scale droplets. In 2008, Fan et al. [27] successfully performed cell sorting by manipulating the droplet using DEP and electrowetting. Singh et al. [28] numerically and experimentally showed the transport and deformation of glycerin droplets using DEP and electrowetting. DEP has great potential in manipulating all kinds of droplets, including liquid metal droplets. According to the features of Gallium-based alloys mentioned before, DEP has potential to provide good access to manipulating EGaInSn droplets quickly and accurately. Thus far, the DEP control for liquid metal droplets has not been reported.

In this work, a microfluidic system was proposed for the generation and sorting of the liquid metal droplets. This system utilized an F-junction generator to generate stable and spherical liquid metal droplets, and then utilized liquid-metal-filled electrodes to perform the active manipulation of these droplets using DEP force. The fabrication of the droplet generators and electrodes is very simple, cheap and convenient, which can also help the integration of these micro devices into the microfluidic system.

## 2. Liquid Metal Based Microfluidic Chip

**Figure 1:**
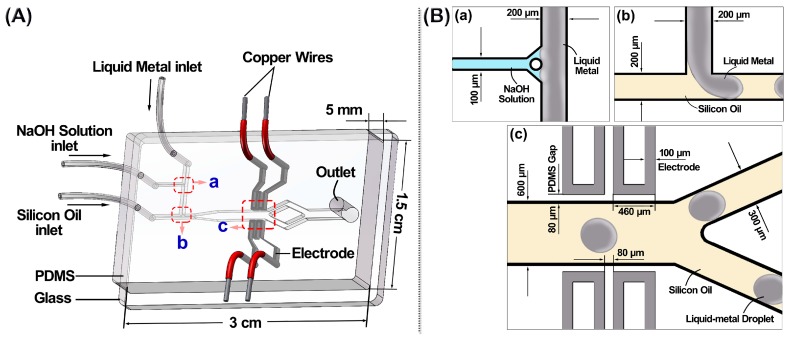
(**A**) Schematic view of a polydimethylsiloxane (PDMS)/glass microfluidic chip; (**B**) Zoom-in schematic of (**a**) junction of NaOH flow and liquid metal flow; (**b**) droplet generation using silicon oil as the continuous phase and deoxidized liquid metal as the dispersed phase; (**c**) Y-shape microchannel to test droplet sorting under the control of two pairs of liquid metal electrodes.

Figure 1A shows the schematic view of a PDMS/glass microfluidic chip for the generation and sorting of liquid metal droplets. The droplet generator utilizes F-junction channels. This F-junction generator is an updated design with a branch channel for the injection of aqueous NaOH solution added to a classical T-junction generator. With the help of NaOH solution, this generator can easily generate liquid metal droplets without oxide layers. To manipulate these generated droplets, the chip utilizes liquid-metal-filled channels as electrodes to induce electric fields across the droplet channel. These electrodes are located symmetrically on both sides of the droplet channel in the same horizontal level, and have no contact with the fluid in the central channel. The PDMS gap between the droplet channel and the electrode channel is 80 μm. Figure 1B shows the specific configuration of the F-junction generator (a and b) and the sorting part (c) in the chip, and also presents the working principle of droplet generation and sorting. When a certain voltage is applied on the liquid metal electrodes, the liquid metal droplets flow towards the target sidewall of the droplet channel under the DEP force**.** The open circle in Figure 1B-a is the PDMS pillar in the branch channel. The pillar can hinder the liquid metal flowing backwards into the branch channel without reducing the contact area between the liquid metal and the NaOH solution.

## 3. Experimental Details

**Figure 2:**
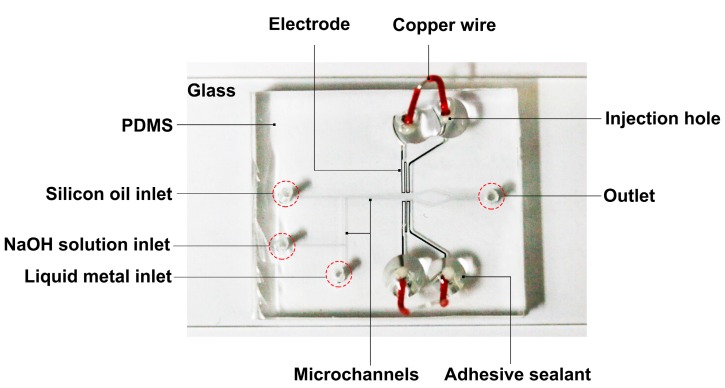
Optical photograph of a PDMS/glass microfluidic chip for liquid metal droplet generation and sorting by liquid metal electrodes.

Figure 2 shows the microfluidic chip for the generation and sorting of liquid metal microdroplets. The chip was fabricated using standard soft-lithography technology. SU-8 2075 (MicroChem Corp., Westborough, MA, USA) was used to fabricate 80 μm high microchannel patterns. Sylgard 184 silicone elastomer (mixture of base and curing agent at a 10:1 ratio by weight, Dow Corning, Midland, MI, USA) was used to fabricate the PDMS slab. By using the plasma treatment, the patterned PDMS slab was irreversibly bonded with a glass slide (75 mm × 52 mm × 1 mm). Ga_75.5_In_24.5_ (75.5 wt % Ga, 24.5 wt % In, melting point: 15.7 °C; Shanxi Zhaofeng Gallium Co., Ltd., Shanxi, China) was injected into the microchannel to fabricate electrodes. A syringe was used to handily inject this liquid metal into electrode channels [9,10]. Copper wires with the diameter of 200 μm were then inserted into the injection holes at all ends of liquid metal channels. To fasten the connection between the wire and the liquid metal inside the electrode channel, an adhesive sealant (705 RTV Silicone Rubber, Kangda Chemical Co., Ltd., Liyang, China) was used to seal the wire joints.

During the experiments, the liquid metal was first deoxidized by the NaOH solution at the junction a (in Figure 1B). Then, the deoxidized liquid metal was cut into microdroplets at the junction b (in Figure 1B) by the silicon oil flow. Finally, the generated liquid metal droplets flowed into a broadened 600 μm wide microchannel, and then were controlled by the liquid metal electrodes and sorted into two 300 μm wide microchannel. Because the chip was only designed to show the mechanism of sorting, to simplify the structure and make the whole system more stable, the two split 300 μm wide microchannels converged into a 600 μm wide microchannel at the end.

Three syringe pumps (LSP10-1B, Longer Precision Pump Co, Ltd., Baoding, China) were used to inject liquid metal, NaOH solution (30 wt %, Beijing Chemical Works, Beijing, China) and silicon oil (Xilong Chemical Co., Ltd., Shantou, China) into the microfluidic system to perform the generation of liquid metal droplets. A high voltage sequencer (HVS448 6000D, LabSmith, Inc., Livermore, CA, USA) was used to offer high voltages for the dielectrophoresis to control liquid metal droplets. A common inverted microscope (100×, COIC, Chongqing) with a CCD camera was used to monitor the movements of the liquid metal droplets.

## 4. Results and Discussion

### 4.1. Microdroplet Generation on an F-Junction Chip

Actually, at first, the classic T-junction generator was used to generate a liquid metal droplet. Without the help of NaOH solution, because of the gallium oxidation, it is hard to generate uniform and continuous droplets by only using silicon oil as the continuous phase and eGaInSn as the dispersed phase. To remove the oxide layer of gallium, a branch channel for the supplement of 30 wt % NaOH solution was added to the T-junction generator before the liquid metal met with the silicon oil at the main intersection point. Thus, instead of the T-junction generator, the “F-junction” generator was used in this work to generate a liquid metal droplet.

**Figure 3:**
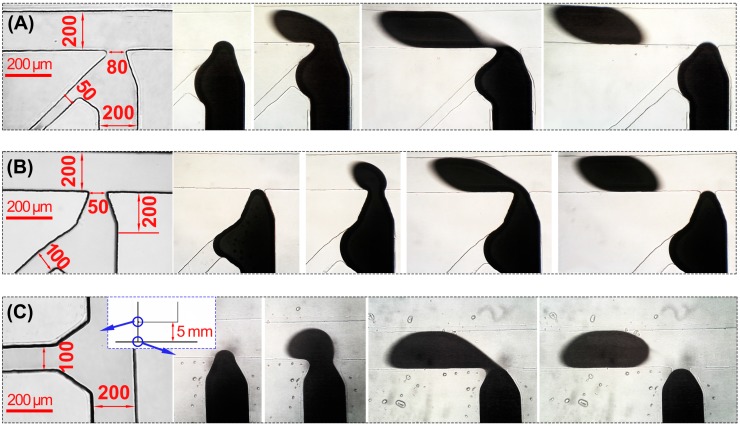
Three designs for droplet generation: (**A**) F_0_-type; (**B**) F_1_-type; (**C**) F_2_-type.

The location of the branch NaOH channel is an important factor for the droplet generation. Three designs (F_0_-type, F_1_-type and F_2_-type, as shown in Figure 3) were considered in this work. As shown in Figure 3, in the first two designs (F_0_-type and F_1_-type), the joint of the NaOH channel and the liquid metal channel is very close to the main joint of the silicon oil channel and the liquid metal channel. In F_0_-type, two joints are merged as one. F_1_-type has a distance of 200 μm between the two joints. In F_2_-type, the distance of the two joints is much longer, 5 mm. The width of the branch NaOH channel in F_0_, F_1_ and F_2_ type are 50 μm, 100 μm and 100 μm respectively. All the fluids were controlled at a flow rate between 2 μL/min and 20 μL/min.

During the droplet generation, the flow rate ratio between the continuous phase and the dispersed phase was all controlled at $\nu_{c}:\;\nu_{d}$ = 3:1, where $\nu_{c}$ and $\nu_{d}$ are the flow rate of the continuous phase and the dispersed phase. In all the three designs, the NaOH solution successfully deoxidized the liquid metal and wrapped the liquid metal with a smooth edge. As shown in Figure 3, because the joint of the NaOH channel is too close to the main joint in F_0_ and F_1_ type, the liquid metal has a trend to flow back into the NaOH branch channel and this trend easily affects the pressure distribution at the main joint for droplet generation, which makes the control very unstable. By comparing F_0_ with F_1_ type in Figure 3, another conclusion is that the width of the branch NaOH channel has no obvious influence on the experimental result. Comparing the results of these three types, F_2_-type shows the best performance and gives stable and perfect drop generation. Thus, F_2_-type is chosen as the structure for droplet generation in the final design.

**Figure 4:**
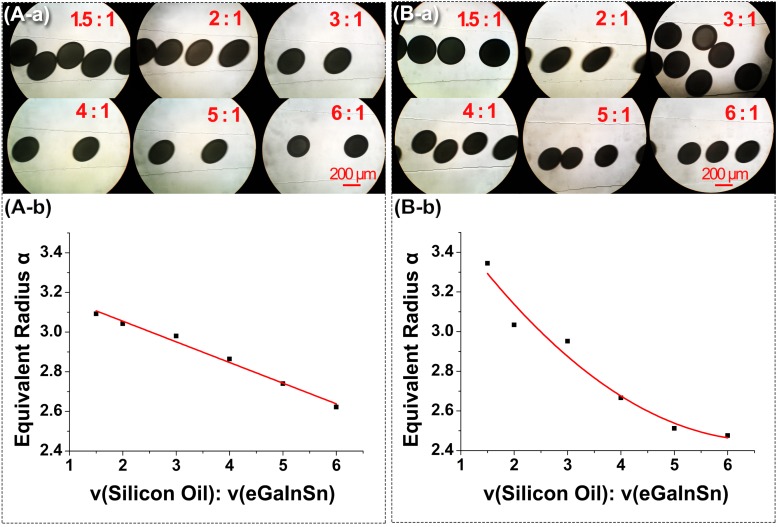
Droplet generation under two conditions. (**A-a**,**A-b**) The flow rate of the silicon oil was kept constant during the experiments; (**B-a**,**B-b**) The velocities of the liquid metal and NaOH solution were kept constant during the experiments.

Another important factor that may affect the droplet generation is the flow rate ratio of silicon oil to liquid metal. Figure 4 shows the droplets generated under different flow rate ratios ranging from 1.5:1 to 6:1. According to the above analysis, F_2_-type was chosen as the chip for the droplet generation. In order to avoid the liquid metal pausing or flowing backwards into the NaOH solution channel, it was necessary to adjust the flow rate of the NaOH solution while changing the flow rate of the liquid metal. During the experiments, considering that the velocity of the liquid metal and NaOH should be comparable to keep the flow well balanced, the flow rate ratio should agree with the formula of $\nu_{d}:\;\nu_{b}\approx w_{d}:w_{b}$, where $\nu_{d}$ is the flow rate of the liquid metal, $\nu_{b}$ is the flow rate of the NaOH solution, $w_{d}$ and $w_{b}$ are the widths of the dispersed phase channel and the branch channel respectively. Figure 4A-a,A-b shows the photographs of the droplets and their dimensionless characteristic parameter under different flow rate ratios when the flow rate of the silicon oil is kept constant. Figure 4B-a,B-b shows the photographs of the droplets and their dimensionless characteristic parameter under different flow rate ratios when the flow rate of the liquid metal and the NaOH solution is kept constant.

The dimensionless characteristic parameter α (equivalent radius) of droplets can be given as

(1) $$\alpha =\frac{1}{w_{mj}}\times \sqrt{\frac{S}{\pi }}$$

where $w_{mj}$ is the width of the main joint with the value of 50 μm, S is the area of droplets. The dimensionless characteristic parameter α of droplets decreases with the increment of the flow rate ratios. According to the formula of $\nu_{d}:\;\nu_{b}\approx w_{d}:w_{b}$, the flow rate of the NaOH solution in the branch channel has to be changed as the flow rate of the continuous phase changes. As shown in Figure 4A, within the range of [1.5, 6], the relation between the droplet size and the flow rate ratio is similar to a linear line. This distribution can help to control the droplet size.

In the F-junction generator, liquid metal microdroplets wrapped with NaOH solution can behave like real liquid with smooth edges. Furthermore, the F-junction chip can also generate controllable sizes of spherical droplets through the changes of flow rate ratios of the continuous phases to the dispersed phases.

### 4.2. Liquid Metal Microdroplets Sorting

**Figure 5:**
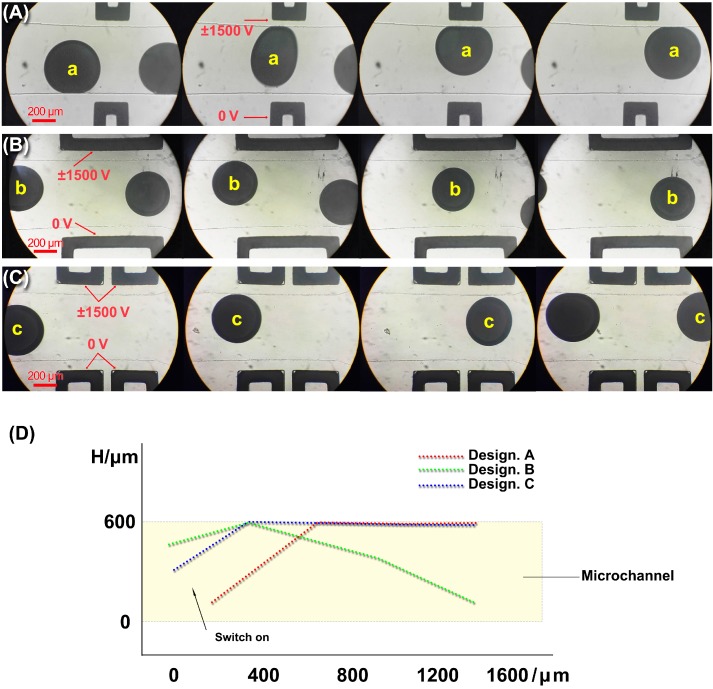
Liquid metal droplet sorting with three different patterns of electrodes; (**A**) Design A; (**B**) Design B; (**C**) Design C; (**D**) Droplet moving paths in Design (**A**–**C**).

To investigate the effect of microelectrode geometry on the liquid metal droplets’ sorting, three designs are demonstrated in this section, as shown in Figure 5, including Design A (a pair of short electrodes, 300 μm long), Design B (a pair of long electrodes, 1000 μm long), and Design C (two pairs of short electrodes, 460 μm long with an 80 μm PDMS gap, actually splitting the long electrode into two). The microchannel for droplets and fluid is 600 μm wide, and the gap between the microchannel and the microelectrodes is 80 μm.

Figure 5A–C shows the deformation of the liquid metal droplets with different electrode designs. The diameter of the droplets was 300~400 μm. Figure 5D shows the moving part of the liquid metal droplet in the three designs. H is measured by using the software of a Carl Zeiss Fluorescence Microscope. The square-wave electric potential was applied on the upper microelectrodes with the high voltage +1500 V and the low voltage −1500 V. The frequency was 1/3 Hz. The voltage applied on the lower electrodes was kept constant at 0 V. After the electric field was switched on, the DEP force caused the droplets to deform and move towards the high electrical field, which was at the corner of the electrodes. Three designs were considered for the droplet sorting as follows.

As mentioned above in Figure 5A, the electric potential is applied on the upper electrode, and the droplet deforms quickly and moves towards the upper sidewall. When the length of the electrodes gets longer, as shown in Figure 5B, the electrode failed to attract the droplet all the time. The droplet moves towards the upper sidewall and then goes down to the lower sidewall. It seems that only the corner of the electrode can attract the droplet and the droplet loses its attraction force once it leaves the corner. If we split the long electrode into two and keep the total length of the electrode the same as shown in Figure 5C (compared to Figure 5B), the electrodes successfully attract the droplet to the upper sidewall all the time again.

When a small spherical droplet of radius a is subjected to a nonuniform electric field E, it gets polarized on the surface under the electric stress due to the difference between the dielectric constants of the droplet and the ambient fluid. The electric stress can deform the droplet and generate a net electric force, which causes the movement of the droplet. This phenomenon is referred to as dielectrophoretics (DEP), and the DEP force can be given by [29]

(2) $$F_{DEP}=2\pi \varepsilon_{m}Re\left[K\left(\omega \right)\right]a^{3}\nabla E_{rms}^{2}$$

where $E_{rms}$ is the root mean square (RMS) value of the electric field, and $\varepsilon_{m}$ is the permittivity of the fluid. The expression above is valid for both the AC and DC electric field. Re[K(ω)] is the real part of the Clausius–Mossotti (CM) factor, which determines the direction of the force. The CM factor determines the extent of polarization given by

(3) $$K\left(\omega \right)=\frac{\varepsilon_{p}^{*}-\varepsilon_{m}^{*}}{\varepsilon_{p}^{*}+2\varepsilon_{m}^{*}}$$

where $\varepsilon_{p}^{*}$ and $\varepsilon_{m}^{*}$ are the frequency-dependent complex permittivity of the droplet and the fluid, and given by

(4) $$\varepsilon^{*}=\varepsilon -j\frac{\sigma }{\omega }$$

ε and σ are the dielectric constant and conductivity, and $j=\sqrt{-1}$, ω is the radian frequency of electric field, respectively.

For liquid metal droplets, Re[K(ω)]≈1, and $F_{DEP}$ is greater than zero. This indicates that the droplet is more likely to move to the direction of the high-intensity electric field, referred to the positive dielectrophoresis. The above expressions are valid when the deformation of the droplet is neglected. However, when it comes to liquid metal droplets, these expressions may not be available because of the droplet deformation. Hence, to explain the movement difference between Figure 5A–C, numerical simulations of the electric field are presented by COMSOL Multiphysics 3.5 without considering the droplet deformation.

The geometry of three electrode patterns is the same as the experimental chips. Due to the high resistivity of PDMS and silicon oil, the electric potentials of ±1500 V and 0 V are applied on the upper and lower electrodes, respectively.

**Figure 6:**
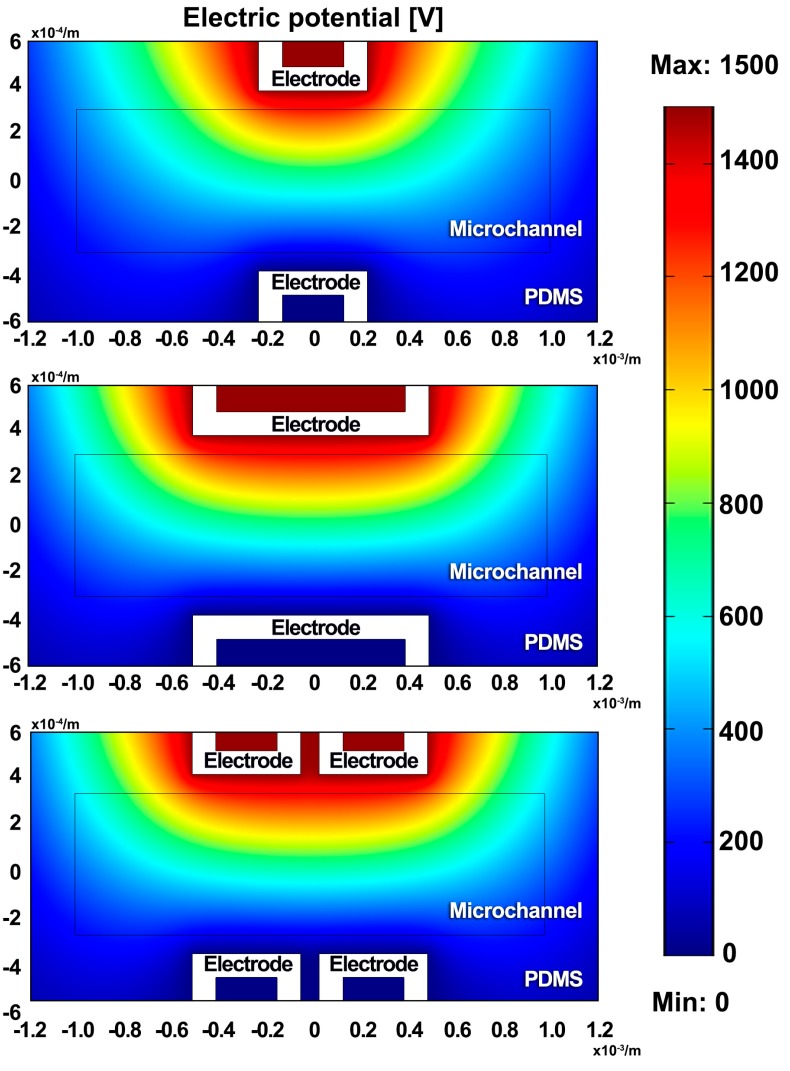
Numerical simulation of an electric potential of 1500 V applied to the upper electrodes of three designs.

**Figure 7:**
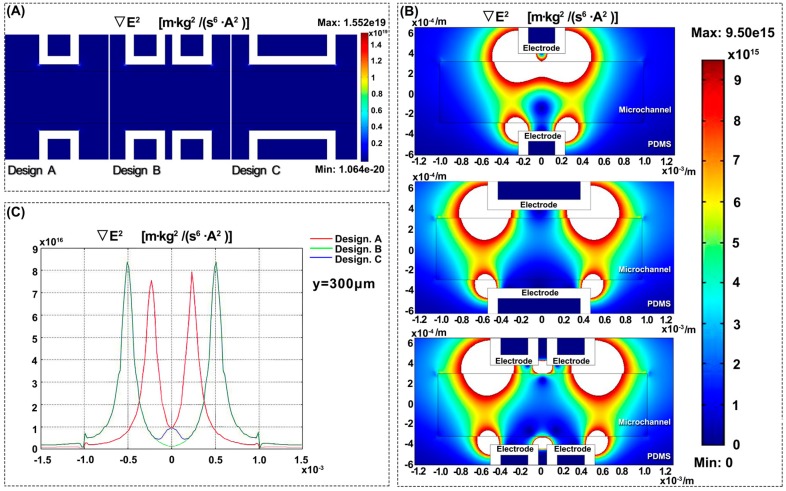
Numerical simulation of $\nabla E^{2}$ for an electric potential of 1500 V applied to the upper electrodes of three designs. (**A**) Full value of $\nabla E^{2}$ in three designs; (**B**) Limiting value of $\nabla E^{2}$ ranging from 0 to 9.50 × 10^15^; (**C**) Value of $\nabla E^{2}$ on the sidewall near the charging electrodes.

Figure 6 shows that the distributions of the electric potential in three designs are almost the same. Figure 7 shows the distribution of the gradient of the electric field square $E_{rms}^{2}$, where $\nabla E_{rms}^{2}$ is used to characterize the dielectrophoretic force. The dielectric constant of the silicon oil [30] and PDMS [31] is 2.76 and 2.5, and their conductivity is 2.5 × 10^−2^ pS/m and 0.345 pS/m, respectively. As shown in Figure 7A, the full distribution of $\nabla E^{2}$ ranges from 1.064 × 10^−20^ to 1.552 × 10^19^ m·kg^2^/(S^6^·A^2^). In order to display the differences between three designs obviously, the value of $\nabla E^{2}$ is limited, ranging from 0 to 9.50 × 10^15^ m·kg^2^/(S^6^·A^2^) in Figure 7B. The white area shows the area with super high $\nabla E^{2}$. In other words, the DEP force in the white area will be fairly large and likely drag the droplet passing through it to its sidewall. It can be seen from Figure 7B that these three designs have almost similar $\nabla E^{2}$ distribution except the area near the electrode corner. The $\;\nabla E_{rms}^{2}$ has a very high value at the electrode corner with the high-intensity electric field. It indicates that the electrode corner has strong DEP attraction to the droplets.

Figure 7C shows the distribution of $\nabla E^{2}$ along the upper sidewall in the three different Designs A, B and C. As shown in Figure 7C, because of the short electrode edge of Design A, the whole edge falls into the white area, which means that the DEP force will drag the droplet to the upper sidewall during the whole process. When the electrode edge gets longer in Design B, the $\nabla E_{rms}^{2}$ at the corner of the electrodes is still strong, but the $\;\nabla E_{rms}^{2}$ near the middle of the electrode edge drops a lot. So, in Design B, the droplet moved to the white area near the corner at first and then left the upper sidewall near the middle of the electrode edge.

Actually, the droplet should be attracted twice by the DEP force at the two corners in Design B. However, the second attraction spot is too close to the split point and the split flow dominates the movement of the droplet at that time. In Design B, the droplet was gradually attracted to the upper wall at the first corner by the first DEP attraction. Because the distance between the two attractions in Design B is too long, the droplet lost the attraction after passing through the first corner and flows to the center of the microchannel again. At this time, the flow began to split and the droplet has a strong trend to go with it. The strong trend with the split flow can be explained as follows. Because of its high density, the liquid metal droplet has a much larger inertia force at the split point compared with the DEP force. The DEP force is strong only when the droplet is close to the corner. That is why, if one wants to successfully keep the droplet “stuck” to the upper wall, one should keep the droplet close to “the corners” all the time. As shown in Figure 5, in Design B, the DEP force of the second corner is very week after the droplet moves away from the upper wall and the split flow takes over the droplet and dominates the rest of its movement.

Things are quite different in Design C. If the long electrode edge in Design B is split into two short electrode edges as shown in Design C, there is a new “white area” caused by the “new” corners appearing near the middle and this “white area” or “new” corners help to keep the droplet from flowing down to the lower sidewall. As mentioned earlier, different from Design B, when the droplet passes through the middle area in Design C, two “new” electrode corners near the middle drag the droplet again and keep the droplet at the upper sidewall.

The results of the numerical simulation agree well with the experimental results from Figure 5. If the length of the electrode is larger than the diameter of the droplet, there will be a ‘blind zone’ (as shown in Design B, the non-white area at the middle of the electrode edge) for DEP force to drag droplets. Splitting one long electrode into several short electrodes will also help in controlling the liquid metal droplet in such structures by making the droplet stick on one side of a channel.

## 5. Conclusions

This work proposes and demonstrates a new liquid metal droplet generator, F-junction. This generator can generate uniform droplets easily. Additionally, a DEP force based sorting structure is designed. In this structure, the liquid metal microchannel is used as micro electrodes to generate DEP force to control the liquid metal droplet. This sorting device has the universality in the active sorting of cells, grains and particles. Furthermore, in the experiments, this device accomplished the target of selectively picking up one or any liquid metal droplet. This work provides handy access to generating and manipulating these neutral liquid metal droplets in nonconductive fluid. The liquid metal droplets combine the advantages of both liquid metal and droplets, having potential in applications of the PCR process [32], drug delivery, thermal probe and so on.

## Supplementary Materials

The following are available online at www.mdpi.com/2072-666X/8/2/39/s1, Video S1–S3: Liquid metal droplet sorting in the F-junction chip, including three patterns of electrodes.
